# Supplementary material for: Investigating the relationship between internal spinal alignment and back shape in patients with scoliosis using PCdare: A comparative, reliability and validation study
Source: PLoS One. 2025 Jul 14;20(7):e0321429. doi: 10.1371/journal.pone.0321429 (PMC12258602; doi:10.1371/journal.pone.0321429)
Supplement: Attachment — Submitted filename: Response to Reviewers.pdf [file pone.0321429.s002.pdf]

Answers to reviewers

Page and line numbers correspond to the Revised Manuscript with Tack Changes

Reviewer #1: The manuscript written by Kaiser et al focuses on using PCdare to improve ease of interpretation for AIS. Overall, the clinical involvement and analysis is interesting, and the research aligns well with the guidelines in PLOS One. However, some essential revisions are needed before it is ready for publication.

1. It would be nice to have a reference supporting the claim that up to 3% of teenagers are affected by AIS (Line 58). Is this in the United States or globally? I specification on this would be helpful for context of AIS cases. Likewise, the authors mention that several studies have reported the incidence of radiation-induced cancer types is elevated in patients with AIS but only cites one reference. Is there more evidence to support the wording of "several"?

Thank you for this helpful observation. The reference supporting the claim that up to 3% of adolescents are affected by AIS was originally cited in the following sentence; however, to improve clarity, we have now added the citation directly after the first mention of this statistic (page 3, line 62). We also clarified that this estimate refers to the global adolescent population by adding the word "globally" (page 3, line 62).

Regarding the use of "several studies" in reference to radiation-induced cancer risk: To strengthen this statement, we have added an additional reference from Luan et al. ("Cancer and mortality risks of patients with scoliosis from radiation exposure: a systematic review and meta-analysis," European Spine Journal, doi: 10.1007/S00586-020-06573-7) that includes a systematic review and meta-analysis, providing broader support for the claim (page 3, line 65).

2. The PCdare software and the overview of what the software does should be explained in the introduction where Kaiser et al is mentioned. Perhaps a brief description of the software should be made after the sentence describing how the software can be used to register 3D surface scans on Line 73. This will really make the method and significance of this software stand out.

Thank you for this helpful suggestion. We have added a short paragraph outlining the essential workflow and functionality of the PCdare software to provide readers with a clearer understanding of its role in the study (page 4, lines 74-80).

3. In regard to the time savings mentioned on Line 73, how much time was saved using such method? Having this specified may help show the significance of this approach compared to traditional methods.

Thank you for this valuable suggestion. While the time savings are detailed in the cited reference, we recognize that including this information directly in the manuscript enhances its clarity. Therefore, we have now explicitly stated that the registration process using our method requires approximately two minutes, compared to the five to ten minutes typically reported in the literature (page 4, lines 82-83).

4. The section written between Lines 101-112 is written out of order in respect to the content in Fig 1. It would naturally flow and read easier with the figure if Study 1 was mentioned first, Study 2, then to Study 3.

Thank you for this thoughtful comment. In principle, we agree that aligning the text with the order of Figure 1 would improve the narrative flow. However, we chose to present the studies in

chronological order (Studies 1–3) to reflect the progression of our research. Importantly, Study 3 incorporates key methodological improvements—such as the use of markers and posture replication—that were informed by the findings of Studies 1 and 2.

Presenting Study 3 first in the detailed explanation allows us to clearly highlight these advancements and then contrast them with the earlier studies, which lacked these features. Starting with Study 1 would require us to repeatedly clarify the absence of these elements, which could disrupt the clarity of the comparison. For this reason, we believe the current structure better supports the reader's understanding of the methodological evolution and core differences between the studies, and we would prefer to retain this order.

5. The authors mentioned on Line 123 that 7 images were used. Among them, 1 was with a normal spine, 2 with mild scoliosis, 3 with moderate, and 1 with severe. Was there a reason for the number of 7 images? And likewise, why were the patient breakdown unequal?

Thank you for these insightful questions. To maintain a concise manuscript, we initially omitted some methodological details. As noted at the end of Section 2.1 (page 9, line 182-191) and in the Results (page 11, line 222-223), we conducted an a priori power and sensitivity analysis. This analysis initially considered variations in the number of raters, images, and repetitions. However, the final numbers were ultimately determined by practical constraints, including clinician availability and the need to limit their time commitment to a manageable level. We have now included these methodological details in the revised manuscript (page 7, lines 131-134).

Regarding the distribution of case severity, the seven images were pseudo-randomly selected to reflect the actual distribution observed in Study 3. We agree that this information is important for transparency and reproducibility and we have now clarified this in the revised manuscript (page 7, lines 138-142).

6. Were there considerations for statistical pairwise comparisons to generate a p value across students and clinicians to show if there is/lack of significance between the two groups for more informative results?

Thank you for this insightful question. If we have correctly understood your concern, the linear mixed-effects model we employed was designed to account for variability across both raters groups, and thus inherently addresses differences between students and clinicians. Given the already extensive number of statistical evaluations included in the manuscript, we chose to limit additional analyses. However, we recognize the value of such comparisons and as stated in the Data Availability section, both the R code and data are publicly available at: https://github.com/mkaisereth/PCdareSoftware. We encourage interested readers to explore the dataset and code for further analysis.

7. It appears that the equations written are in coding notation. If able, it would be more legible to present such calculations with the mathematical formulas, and referencing the packages/software used if any. I notice R is mentioned on Line 160, but not prior, which may be associated with the confusion.

Thank you for this helpful comment. You are correct—we use coding-style notation to align with the structure of our publicly available R scripts. Since we aim to promote transparency and reproducibility, we prefer to retain this notation for consistency with the shared codebase.

That said, we agree that introducing R earlier in the manuscript would help orient the reader and reduce potential confusion. We have now added this clarification earlier in the Methods section (page 8, line 157-158).

8. Scale bar for the images in Fig 3. is missing.

Thank you for this comment, we have now added a scale bar to Fig 3.

9. Fig 5,6, and 7 should have all measurements plotted on the box plot to make it clear to the readers the variation of each reading, rather than the outliers only. It would further inform the type of distributions and clusters that are hidden in this current format.

Thank you for this valuable suggestion. We have updated Figures 5, 6, and 7 to include all individual measurements on the boxplots using a swarmchart-style overlay. This provides a clearer visualization of the distribution, variation, and potential clustering within each dataset, as recommended.

10. Minor aesthetics comments on Fig 6 and 7. It appears Fig 6 is a screenshot, with the plot (left one) appearing to be clicked on prior to saving. Between these two figures, I see a minor dashed line in some plots, but not in others. What does this mean?

Thank you for pointing this out. You are correct—Figure 6 contained an aesthetic error. We have corrected this and updated the figure accordingly.

Regarding the dashed lines visible in some plots: these are a feature of MATLAB's 'DataLim' behaviour, which occurs when the y-axis is limited and outliers are plotted at the boundary using dashed lines. This approach helps to focus the figure on the most relevant data range while still indicating the presence of extreme values. We have now added a brief explanation of this behaviour to the captions of Figures 6 and 7 for clarity.

11. On Line 315-316, the authors mention how "a high correlation coefficient is to be expected even in patients with scoliosis." Perhaps another sentence explicitly stating the reasoning may be informative to readers not fully aware of the field.

Thank you for this helpful suggestion. We have added a sentence to clarify the reasoning behind the expectation of a high correlation coefficient, even in patients with scoliosis (page 17, line 345-346).

12. There are numerous areas that mention future work in the Discussion. (e.g., Lines 278, 290, 297, 301, 324, then the final paragraph in the Discussion). It may be easier to read if all mentions of future work is compartmentalized into one paragraph, perhaps at the end of the Discussion section into the paragraph along Lines 325-331.

Thank you for the suggestion. We have consolidated all mentions of future work into a single paragraph at the end of the Discussion section (page 17, line 355-359) to improve readability.

Reviewer #2: The authors highlighted the PCdare research software usage through 3 experimental studies, and investigated its reliability as a drawing tool for internal spinal alignment(ISL) in this current work. The experimental setup was well explained for the all 3 studies and it managed to help readers understand the effect of the markers and posture measurement. The approach, methodology and results support the objective of the work. The discussion also proposes explanations for the encountered errors with possible expansions in future work.

It should be noted that code is made available but dataset is not shared but promised to be shared in a future data publication.

Thank you for this comment. All code and the IIR study data are now publicly available (https://github.com/mkaisereth/PCdareSoftware/tree/main/IIR), allowing full replication of the statistical analyses. The raw data (X-ray images and 3D surface scans) will be shared in a separate data publication, which is already prepared and will be submitted shortly, however, we have now already uploaded the X-ray images as JPG images and the 3D surface scans for Study 3.

There are few points that may help readers understand the reasoning behind some of the steps taken in the analysis, the first of which is the reasoning for the selection of 7 images only from the study 3 with 30 subjects available?,

Thank you for these insightful questions. To maintain a concise manuscript, we initially omitted some methodological details. As noted at the end of Section 2.1 (page 9, line 182-191) and in the Results (page 11, line 222-223), we conducted an a priori power and sensitivity analysis. This analysis initially considered variations in the number of raters, images, and repetitions. However, the final numbers were ultimately determined by practical constraints, including clinician availability and the need to limit their time commitment to a manageable level. We have now included these methodological details in the revised manuscript (page 7, lines 131-134).

another followup explanation is for the reason for the class distribution [1,2,3,1]? is this a stratified random selection? or the two extreme cases (normal and severe) are easily examined and drawing of ISL poses no challenge for example?, it is not clear as to why such distribution is made assuming all 30 subjects have AIS.

Regarding the distribution of case severity, the seven images were pseudo-randomly selected to reflect the actual distribution observed in Study 3. We agree that this information is important for transparency and reproducibility, and we have now clarified this in the revised manuscript (page 7, lines 138-142).

The use of median can be understood after examining the box plots but it would be clearer to readers if it is explained to understand the justification.

Thank you for this helpful comment. To improve clarity, we have added all individual data points to the box plots in Figures 5–7, allowing readers to better visualize the underlying distributions.

Also, it might help if the reasons why images from study 1 and 2 are not used in the inter/intrarater reliability testing are explained, especially study 2 as it shows similar performance when compared to study 3(Table 5).

Thank you for this question. The reason we used only images from Study 3 for the inter- and intra-rater reliability testing is that it provided the highest data quality, with the smallest alignment errors. This allowed us to minimize the influence of confounding factors and focus more accurately on rater performance. We have now added a brief explanation to the manuscript (page 7, line 139-141).

Some extra details for the study protocol, what are the selection criteria for the participants and annotator?, would additional annotators provide better understanding of variability?

Thank you for this suggestion, we added a Table (Table 1) with inclusion and exclusion criteria and demographics (page 5, line 111).

Regarding additional annotators, we agree, which we already mention at the end of the discussion section: “Expanding the IIR study to involve a larger number of clinicians using PACS would offer greater insight into the reliability of the validation” (page 18, line 368-369)

Reviewer #3: This is an automated report for PONE-D-25-09339. This report was solicited by the PLOS One editorial team and provided by ScreenIT.
ScreenIT is an independent group of scientists developing automated tools that analyze academic papers. A set of automated tools screened your submitted manuscript and provided the report below. Each tool was created by your academic colleagues with the goal of helping authors. The tools look for factors that are important for transparency, rigor and reproducibility, and we hope that the report might help you to improve reporting in your manuscript. Within the report you will find links to more information about the items that the tools check. These links include helpful papers, websites, or videos that explain why the item is important. While our screening tools aim to improve and maintain quality standards they may, on occasion, miss nuances specific to your study type or flag something incorrectly. Each tool has limitations that are described on the ScreenIT website. The tools screen the main file for the paper; they are not able to screen supplements stored in separate files. Please note that the Academic Editor had access to these comments while making a decision on your manuscript. The Academic Editor may ask that issues flagged in this report be addressed. If you would like to provide feedback on the ScreenIT tool, please email the team at ScreenIt@bih-charite.de. If you have questions or concerns about the review process, please contact the PLOS One office at plosone@plos.org.

1. Ethics

Ethics statements are found on page 1 and Methods section

2. Inclusion and exclusion criteria

Now added in Table 1

3. Flow charts and attrition

Not required for detailed study protocols; also Fig1

4. Sex as a biological variable

Now added in Table 1

5. Subject demographics

Now added in Table 1

6. Randomization

Pseudo-randomization (page 7, line 138-146)

7. Blinding

n.a. / Shuffled randomly (page 7, line 145-146)

8. Power analysis

First subsection in Methods and first subsection in Results

9. Replication

3 repetitions (page 7, line 145)

10. Open code

Open code available, see data availability statement

11. Open data

Open data available, see data availability statement

12. Self-acknowledged limitations

Many limitations can be found in the Discussion section, i.e. last two paragraphs about future work

13. Conflict of interest

Now added on page 1

14. Funding statement

Now added on page 1

15. Registration statement

n/a

16. No bar graphs of continuous data

fulfilled

17. No rainbow color maps

fulfilled
